# A One-Season Prospective Study of Illnesses, Acute, and Overuse Injuries in Elite Youth and Junior Track and Field Athletes

**DOI:** 10.3389/fspor.2019.00013

**Published:** 2019-09-10

**Authors:** Paul Carragher, Alan Rankin, Pascal Edouard

**Affiliations:** ^1^Sport Ireland Institute, National Sports, Dublin, Ireland; ^2^SportNI Sports Institute, Jordanstown, United Kingdom; ^3^Sports Medicine NI, Belfast, United Kingdom; ^4^Inter-University Laboratory of Human Movement Science (LIBM EA 7424), University of Lyon, University Jean Monnet, Saint Etienne, France; ^5^Sports Medicine Unit, Department of Clinical and Exercise Physiology, Faculty of Medicine, University Hospital of Saint-Etienne, Saint-Etienne, France; ^6^Medical Commission, French Athletics Federation (FFA), Paris, France; ^7^Swiss Olympic Medical Center, Centre de Médecine du Sport, Division de Médecine Physique et Réadaptation, Centre Hospitalier Universitaire Vaudois, Lausanne, Switzerland; ^8^European Athletics Medical and Anti-Doping Commission, European Athletics Association (EAA), Lausanne, Switzerland

**Keywords:** epidemiology, track and field, top-level athletes, sports injury prevention, health protection

## Abstract

**Background:** In high-level adult athletes, injury incidences and characteristics have been reported during international championships and during one season. Youth track and field athletes are also exposed to injury risk, although less information is available on this specific population, as well as on illness risk.

**Aim:** To determine the prevalence of health problems (i.e., illnesses, acute, and overuse injuries) in high level Youth and Junior Track & Field athletes.

**Method:** During the 2015–16 athletics season (30 weeks from December 2015 to July 2016), we conducted a prospective cohort study on a population of Youth and Junior Irish national level athletes, during which athletes were asked to complete a weekly web-based questionnaire (Oslo Sports Trauma Research Center questionnaire on health problems) regarding their health problems.

**Results:** A total of 70 athletes participated (37 male and 33 female athletes), with an average weekly response rate of 71%. The average weekly prevalence for all athletes was 27% (95%CI 17 to 38%) for all health problems, and 11% (95%CI 3 to 18%) for substantial health problems. Average prevalence varied significantly between endurance and explosive disciplines: a higher prevalence of all and substantial health problems and all and substantial overuse injuries was found in endurance disciplines. A higher prevalence of acute injuries was found in explosive disciplines. Characteristics of acute and overuse injuries differed according to sex and discipline: hamstring strain/cramps/spasms was the main injuries in explosive disciplines, and knee tendinopathy and lower leg strain/cramp/spasms in endurance disciplines, trunk cramps/spasms being frequent in both disciplines. Upper respiratory tract problems were the most commonly reported illnesses regardless of sex and disciplines.

**Conclusion:** This study provides important information regarding the extent of health problem in Youth and Junior track and field athletes. This could help orient injury prevention measures. For injuries, it should be focused on muscle injuries, especially located on the hamstring, calf, and trunk. For illness, prevention measures could include: screening tests for airway problems, but also general illness prevention measures (e.g., drinking regularly, eating “safe” food, regular hand washing, decreasing contact with sick people, avoiding dehydration).

## Introduction

The practice of track and field (athletics) can lead to a risk of injuries that negatively impact the athlete's participation in sport, daily life and/or physical integrity (Edouard et al., 2011, 2015a). In high-level adult athletes, injury incidences, and characteristics have been reported during international championships (Feddermann-Demont et al., 2014; Edouard et al., 2015b) and during one season (D'Souza, 1994; Bennell and Crossley, 1996; Jacobsson et al., 2013). Youth track and field athletes are also exposed to injury risk, although less information is available on this specific population (Watson and Dimartino, 1987; D'Souza, 1994; Edouard et al., 2012; Jacobsson et al., 2012, 2013). High-level youth athletes are potentially exposed to high training load (intensity and volume) and competition pressure. However, to our knowledge, only two studies reported results on the injury rates and characteristics in youth high-level athletics (Jacobsson et al., 2012, 2013). The only prospective study to consider this specific population found the proportion of injured athletes was similar to that of adult athletes (e.g., ≈60%), while injury characteristics differed (Jacobsson et al., 2013). There is thus a need for increasing the knowledge on this specific population, given (i) the lack of data available (Steffen and Engebretsen, 2010), (ii) the need for age specific data, as results from adult studies cannot be extrapolated to this population, (iii) to examine the health benefits/risk profile of high-level athletics practice in youth athletes (e.g., injuries and their potential long-term sequelae; Moseid et al., 2018), and (iv) to guide injury prevention strategies.

Illness also represents a health problem that could be caused by sport participation (i.e., heat-related or gastro-enteritis problems; Pluim et al., 2016; Moseid et al., 2018; Edouard et al., 2019), can also increase the risk of subsequent injuries (Timpka et al., 2017), and lead to a decrease in sport participation or performance. Therefore, when talking about athletes' health, it is of interest to also collect such data to have a more complete view of health issues. Hence, to our knowledge, no data has been published in youth track and field athletes, although previously reported in elite junior Tennis (Pluim et al., 2016), and in a cohort of youth elite athletes from several sports (Moseid et al., 2018).

In this context, the aim of the study was to determine the prevalence of injuries and illnesses in high level Youth and Junior track and field athletes.

## Methods

### Study Design

During the 2015–16 athletics season (from December 2015 to July 2016), we conducted a prospective cohort study on a population of Youth and Junior national level track and field athletes from the Athletics Ireland High Performance Programme (Athletics Ireland Athlete Carding Scheme (AIACS), http://www.athleticsireland.ie/high-performance/carding/). During the 30-week period, athletes were asked to complete a weekly web-based questionnaire regarding their health conditions. The study was approved by the Saint-Etienne University Hospital Ethical Committee (IORG0004981).

### Population

At the beginning of the 2015–16 athletics season, Athletics Ireland (http://www.athleticsireland.ie) provided the main investigator (PC) a list of Youth (Under 18) and Junior (Under 20) level athletes (aged from 16 to 19 years) who had obtained performances of a sufficient standard to qualify them for AIACS. The AIACS selection process requires athletes to achieve set performance criteria stated within a selection policy to be considered for membership to the programme.

All the athletes on the AIACS, and their parents when minors, were invited by email to participate in the injury and illness surveillance study. Athlete consent (and parental consent when athletes were minors) for the survey and the data being used for both surveillance and research purposes was obtained upon admission to the Carding Scheme. Athletes were included if they were registered with Athletics Ireland, had no contraindication for athletics participation, appeared on the list of the AIACS, consented to participate, were able to read and reply to questions in English, and had suitable internet access.

### Data Collection Procedure

At the beginning of the 2015–16 athletics season, baseline data (i.e., sex, age, and discipline) was collected. Disciplines were then grouped into explosive disciplines (sprints, hurdles, throws, jumps, combined events) and endurance disciplines (middle and long distance running, race walking) (Timpka et al., 2017).

During the 30 weeks of the surveillance study, all injury, and illness were recorded by the Athletics Ireland Physiotherapist responsible for the group (PC). An email was sent to all included athletes at the start of each week, with a link to complete an online questionnaire (SurveyMonkeyInc, San Mateo, California, USA). If the questionnaire was not completed, an automated reminder was sent after 24 h and then 48 h after the initial email. The Oslo Sports Trauma Research Center questionnaire on health problems was used to collect data (Clarsen et al., 2013, 2014), which includes four questions on the consequences of health problems on sports participation, training volume, sports performance, and perceived pain. This questionnaire and methods have been reported to be used with success in population of youth athletes in elite junior tennis (Pluim et al., 2016), in adolescent elite orienteerers (Von Rosen et al., 2016), in youth football players (Leppänen et al., 2019), in junior handball players (Aasheim et al., 2018), and in youth elite athletes in multiple sports (Moseid et al., 2018), and appears of interest in sports who have limited access to medical personnel (e.g., athletics; Edouard et al., 2014; Leppänen et al., 2019). Questionnaire responses were reviewed and collated on a weekly basis by the Athletics Ireland Physiotherapist. If the athlete answered the minimum score for each question (full participation without problems/no training reduction/no performance reduction/no symptoms) no further action was taken. If the athlete reported anything other than the minimum value for any question, follow-up was carried out by email, telephone or consultation by the Athletics Ireland Physiotherapist. Using this information, the Athletics Ireland Physiotherapist classified each problem as an illness, acute injury, or overuse injury.

In accordance with the classification system described in the consensus statement for epidemiological studies in athletics (Timpka et al., 2014), health problems were classified as injuries if they were disorders of the musculoskeletal system or concussions, and as illnesses if they involved other body systems such as (but not limited to) the respiratory system, the digestive system and the neurological system, as well as non-specific/generalized, psychological and social problems. Injuries were further categorized into acute and overuse injuries: acute injuries were defined as those whose onset could be linked to specific injury event, whereas overuse injuries were those that could not be linked to a clearly identifiable event (Fuller et al., 2006; Jacobsson et al., 2013). All these classifications were made by the Athletics Ireland Physiotherapist based on the interview and/or physical examination when needed. For all forms of health problems, substantial problems were defined as those leading to moderate or severe reductions in training volume, or moderate or severe reductions in sports performance, or complete inability to participate in sport (i.e., problems where athletes selected option 3, 4, or 5 in either Question 2 or 3). Injuries and illnesses were classified according to locations, types, and severities as described in the consensus statement for epidemiological studies in athletics (Timpka et al., 2014). Locations were grouped into head, trunk, upper extremity, and were detailed for the lower extremity. Diagnoses were the combination of location and type for each injury.

According to Clarsen et al. (2013), in cases where the same diagnosis was interspersed with periods of apparent recovery an effort was made to determine if the cases were exacerbations of unresolved problems or recurrences of fully recovered problems. Illnesses were treated in a similar fashion with repeated episodes of chronic conditions treated as a single case for the purposes of analysis. Data collected in the first week were not included in the summary measures, as per previous recommendations (Clarsen et al., 2013).

### Statistical Analysis

Means and corresponding standard deviations (SD) were calculated for baseline demographics. Potential differences in baseline demographics between male and female athletes and between endurance and explosive disciplines were tested with *t*-tests for continuous variables and Chi^2^ statistics for dichotomous variables.

The response rate was calculated for each week by dividing the number of responders by the number of included athletes, and averaged for the whole study period.

The proportion of athletes presenting with at least one health problem was calculated for all health problems, and separately for illness, acute and overuse injury. Comparisons were performed between (i) female *vs*. male athletes (male being the reference) and (ii) explosive vs. endurance disciplines (explosive being the reference) for athletes' proportion using relative risk (RR) with 95%CI.

According to Clarsen et al. (2014), prevalence measures were calculated for all and substantial health problems, illnesses, injuries, overuse injuries and acute injuries for each week that the project was conducted. This was performed by dividing the number of athletes reporting any form of problem by the number of questionnaire respondents. All prevalence measures were presented as averages, with 95% confidence intervals (95% CI). Characteristics of health problems were presented using descriptive analysis (as frequencies). Comparisons of the average prevalence and of the health problem characteristics were then made between (i) female vs. male athletes and (ii) endurance vs. explosive disciplines using Chi^2^-tests and Bonferroni correction was used to control for multiple tests. Data were processed using Excel software. Significance was accepted at *p* < 0.05.

## Results

### Population

Among the 76 athletes selected and listed for being members of the Athletics Ireland's High Performance Carding System, six athletes declined to participate. A total of 70 (92%) athletes gave their consent and were included in the present study at the start the 2015–16 athletics season (December 1, 2015), consisting of 37 male and 33 female athletes, without significant sex-related differences in the mean age and athletics disciplines distribution (Table 1). The distribution of all the 70 athletes in disciplines was:

-In male athletes: middle distance (18.6%), long distance (7.1%) or race walking (1.4%) for endurance disciplines, and sprints (14.3%), hurdles (2.9%), jumps (1.4%), throws (5.7%), or combined events (1.4%) for explosive disciplines;
-In female athletes: middle distance (5.7%), long distance (2.9%) or race walking (0.0%) for endurance disciplines; and sprints (17.1%), hurdles (7.1%), jumps (2.9%), throws (8.6%), combined events (2.9%) for explosive disciplines.

There were no significant differences between male and female athletes in mean age (*p* > 0.05) and in the distribution of athletics disciplines (Chi2 = 9.4; *p* > 0.05).

**Table 1:** Number (percentage) of athletes included in the study, and number (percentage) of athletes presenting at least one health problem during the 30-week study period according to sex and discipline groups.

	**All athletes**						**Male athletes**						**Female athletes**					
	**Total**		**Endurance**		**Explosive**		**Total**		**Endurance**		**Explosive**		**Total**		**Endurance**		**Explosive**	
**Athletes**																		
n (% of all athletes)	70	(100.0)	25	(35.7)	45	(64.3)	37	(52.9)	19	(27.1)	18	(25.7)	33	(47.1)	6	(8.6)	27	(38.6)
Age (mean (SD))	17.1	(0.8)	17.3	(0.7)	16.9	(0.9)	17.2	(0.8)	17.3	(0.7)	17.1	(0.9)	16.9	(0.8)	17.3	(0.5)	16.8	(0.9)
**Athletes with at least one health problem [n (%)]**																		
All health problems	61	(87.1)	18	(72.0)	43	(95.6)	30	(81.1)	13	(68.4)	17	(94.4)	31	(93.9)	5	(83.3)	26	(96.3)
Illness	42	(60.0)	11	(44.0)	31	(68.9)	18	(48.6)	8	(42.1)	10	(55.6)	24	(72.7)	3	(50.0)	21	(77.8)
Injury	54	(77.1)	17	(68.0)	37	(82.2)	28	(75.7)	12	(63.2)	16	(88.9)	26	(78.8)	5	(83.3)	21	(77.8)
Acute injury	31	(44.3)	7	(28.0)	24	(53.3)	16	(43.2)	6	(31.6)	10	(55.6)	15	(45.5)	1	(16.7)	14	(51.9)
Overuse injury	37	(52.9)	13	(52.0)	24	(53.3)	19	(51.4)	9	(47.4)	10	(55.6)	18	(54.5)	4	(66.7)	14	(51.9)

**Figure 1:**
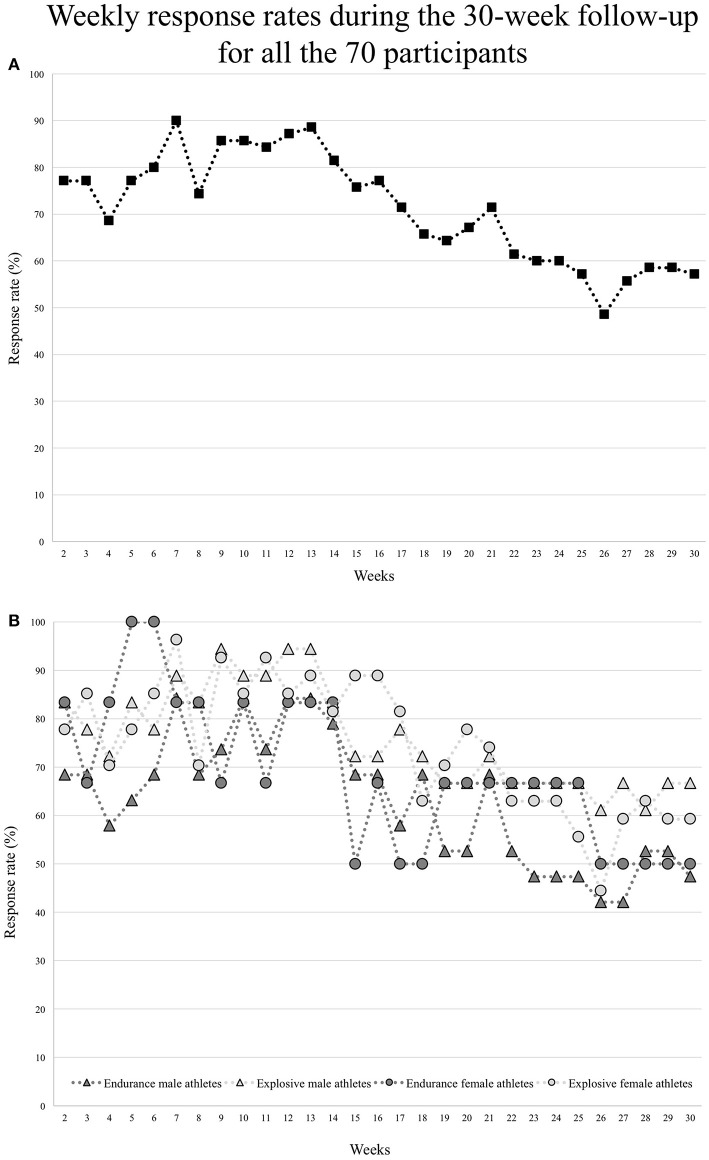
Weekly response rates during the 30-week follow-up for **(A)** all the 70 participants, and for **(B)** endurance and explosive male and female athletes separately.

Over the 30-week period, the average weekly response rate to the health questionnaires was 71.3 ± 11.6 %, without significant differences with regards to sex and disciplines (Table 2 and Figure 1). The individual response rate range from 0 to 100% over the 30-week period: 53 (76%) athletes had a response rate higher than 50%, including 17 (24%) athletes completed all the requested questionnaires (response rate = 100%) and 21 (30%) athletes between 85 and 99% (note that two male athletes replied only to the first questionnaire).

**Table 2:** Average weekly prevalence (with 95% confidence intervals) during the 30-week observation period of all health problems and substantial health problems reported, as well as for subcategories of illness, acute and overuse injury in each subgroup of sex and disciplines.

	**All athletes**			**Male athletes**			**Female athletes**		
	**Total**	**Endurance**	**Explosive**	**Total**	**Endurance**	**Explosive**	**Total**	**Endurance**	**Explosive**
Athletes [n (%)]	70 (100.0)	25 (35.7)	45 (64.3)	37 (52.9)	19 (27.1)	18 (25.7)	33 (47.1)	6 (8.6)	27 (38.6)
Response rate [mean (SD)]	71.3 (11.6)	64.4 (12.5)	75.1 (11.6)	69.2 (11.6)	63.0 (13.3)	75.9 (10.4)	73.6 (12.5)	69.0 (15.3)	74.6 (13.4)
All health problems	27.3 (16.9 to 37.8)	32.7 (21.7 to 43.7)^*^	24.7 (14.6 to 34.9)^*^	25.6 (15.4 to 35.8)	27.4 (16.9 to 37.8)^$^	24.2 (14.2 to 34.2)	29.0 (18.4 to 39.6)	47.1 (35.4 to 58.8)^$^^*^	25.2 (15.0 to 13.0)^*^
Illness	6.8 (0.9 to 12.7)	6.9 (0.9 to 12.8)	6.8 (0.9 to 12.7)	5.6 (0.2 to 11.0)	5.8 (0.3 to 11.2)	5.6 (0.2 to 11.0)	8.0 (1.6 to 14.3)	8.9 (2.2 to 15.5)	7.6 (1.4 to 13.9)
Injury	20.5 (11.0 to 30.0)	25.9 (15.6 to 36.1)^*^	17.9 (8.9 to 26.9)^*^	20.0 (10.6 to 29.3)	21.6 (12.0 to 31.3)^$^	18.6 (9.5 to 27.7)	21.0 (11.5 to 30.6)	38.3 (26.9 to 49.7)^$^^*^	17.5 (8.6 to 26.5)^*^
Acute injury	7.5 (1.3 to 13.7)	4.3 (−0.5 to 9.0)^*^	9.0 (2.3 to 15.7)^*^	7.5 (1.3 to 13.6)	5.5 (0.1 to 10.8)	9.3 (2.5 to 16.1)	7.4 (1.3 to 13.6)	0.9 (−1.3 to 3.0)^*^	8.8 (2.2 to 15.5)^*^
Overuse injury	13.0 (5.1 to 20.9)	21.6 (12.0 to 31.2)^*^	9.0 (2.3 to 15.6)^*^	12.5 (4.8 to 20.3)	16.2 (7.5 to 24.8)^$^^*^	9.3 (2.5 to 16.1)^*^	13.6 (5.6 to 21.6)	37.4 (26.1 to 48.7)^$^^*^	3.9 (−0.6 to 8.4)^*^
Substantial health problems	10.6 (3.4 to 17.8)	17.6 (8.7 to 26.6)^*^	7.4 (1.3 to 13.5)^*^	11.3 (3.9 to 18.7)	15.6 (7.1 to 24.1)^*^	8.0 (1.6 to 14.3)^*^	9.8 (2.8 to 16.8)	22.8 (13.0 to 32.6)^*^	7.0 (1.0 to 13.0)^*^
Illness	2.1 (−1.3 to 5.4)	2.1 (−1.3 to 5.4)	2.1 (−1.3 to 5.5)	2.2 (−1.2 to 5.7)	1.8 (−1.3 to 5.0)	2.7 (−1.1 to 6.6)	1.9 (−1.3 to 5.1)	2.6 (−1.1 to 6.3)	1.8 (−1.3 to 4.8)
Injury	8.4 (1.9 to 14.9)	15.4 (6.9 to 23.8)^*^	5.1 (0.0 to 10.3)^*^	9.2 (2.4 to 15.9)	13.6 (5.5 to 21.6)^*^	5.6 (0.2 to 11.0)^*^	7.5 (1.3 to 13.7)	20.2 (10.8 to 29.6)^*^	4.8 (−0.2 to 9.8)^*^
Acute injury	3.4 (−0.9 to 7.6)	2.6 (−1.1 to 6.3)	3.7 (−0.7 to 8.2)	3.5 (−0.8 to 7.7)	3.5 (−0.8 to 7.8)	3.5 (−0.8 to 7.8)	3.2 (−0.9 to 7.3)	0.0 (0.0 to 0.0)	3.9 (−0.6 to 8.4)
Overuse injury	5.0 (−0.1 to 10.1)	12.8 (5.0 to 20.7)^*^	1.4 (−1.4 to 4.2)^*^	5.7 (0.3 to 11.2)	10.1 (3.0 to 17.1)^$^^*^	2.1 (−1.3 to 5.5)^*^	4.3 (−0.4 to 9.1)	20.2 (10.8 to 29.6)^$^^*^	0.9 (−1.3 to 3.1)^*^

$ *Significant difference between male and female athletes*.

* *Significant difference between endurance and explosive disciplines*.

Among the 70 included athletes, 61 (87.1%) presented with at least one health problem during the 30-week period: 60.0% (*n* = 42) at least one illness, 44.3% (*n* = 31) at least one acute injury, and 52.9% (*n* = 37) at least one overuse injury (Table 1). From these 61 athletes, 13.1% of athletes presented with only one health problem, 26.2% two, 19.7% three, 21.3% four, 16.4% five, and 1.6% six and seven health problems during the study period.

The proportion of athletes was significantly higher in female than male athletes for health problems (RR = 1.27, 95% CI 1.11 to 1.47) and for illnesses (RR = 1.64, 95% CI 1.13 to 2.40); we reported no other sex-related differences for acute and overuse injuries, or in endurance and explosive disciplines (Table 1).

The proportion of athletes with all health problems was significantly higher in explosive than endurance disciplines for all the 70 athletes (RR = 1.33, 95% CI 1.03 to 1.71); we reported no other discipline-related differences for illnesses, acute and overuse injuries and in male and female athletes (Table 1).

### Prevalence of Health Problems

**Figure 2:**
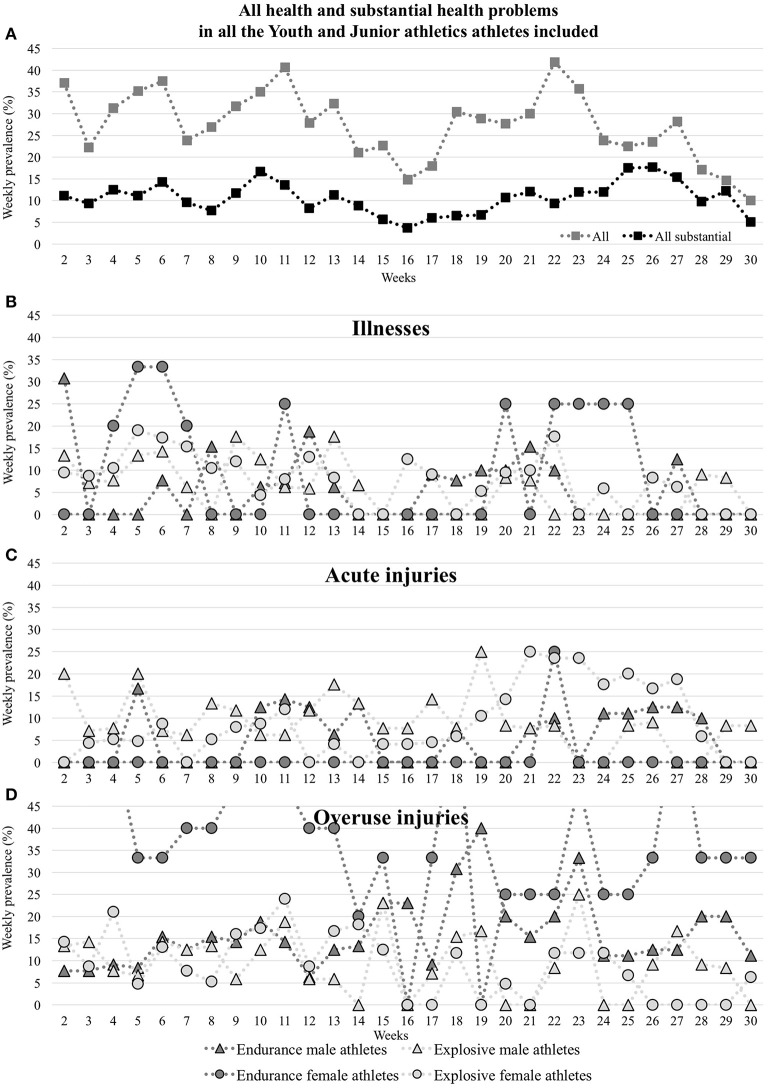
Weekly prevalence of health problems [all and substantial health problems **(A)**, illnesses **(B)**, acute injuries **(C)** and overuse injuries **(D)**] reported during the 30-week follow-up period.

The average weekly prevalence for all athletes was 27.3% (95% CI 16.9 to 37.8%) for all health problems, and 10.6% (95% CI 3.4 to 17.8%) for substantial health problems (Table 2 and Figure 2).

The average weekly prevalence of all and substantial health problems, illness, injuries, acute injuries, and overuse injuries did not significantly vary between male and female athletes when considering all and explosive disciplines. However, in considering endurance disciplines alone, there was a significant difference between male and female athletes for all health problems, injuries, overuse injuries, and substantial overuse injuries (*p* < 0.0001; Table 2 and Figure 2).

The average weekly prevalence of all and substantial health problems, injuries, acute injuries and overuse injuries significantly varied between endurance and explosive disciplines, while not for illnesses and substantial acute injuries (Table 2 and Figure 2).

### Illnesses

A total of 74 illnesses were reported by 42 athletes. 52.4% presented with one illness, 26.2% two, 14.3% three and 7.1% four illnesses. The proportion of athletes with at least one illness was significantly higher in female than male athletes (RR = 1.64, 95% CI 1.13 to 2.40), without other sex-related or discipline-related differences (Table 1).

The average weekly prevalence of illnesses and substantial illnesses was not significantly different between sex and disciplines (Table 2).

Most reported illnesses affected the upper respiratory tract (73.0%), and led to 1 week (68.9%), or between 2 or 3 weeks of absence in sport (31.1%) (Table 3).

**Table 3:** Characteristics of reported illnesses (results are presented in percentage of illnesses per categories of sex and discipline).

	**All athletes**			**Male athletes**			**Female athletes**		
	**Total** ***n* = 74**	**Endurance** ***n* = 22**	**Explosive** ***n* = 52**	**Total** ***n* = 30**	**Endurance** ***n* = 15**	**Explosive** ***n* = 15**	**Total** ***n* = 44**	**Endurance** ***n* = 7**	**Explosive** ***n* = 37**
**Affected system**									
Upper respiratory tract	73.0	81.8	69.2	73.3	86.7	60.0	72.7	71.4	73.0
Lower respiratory tract	9.5	4.5	11.5	6.7	6.7	6.7	11.4	0.0	13.5
Gastrointestinal	10.8	9.1	11.5	16.7	6.7	26.7	6.8	14.3	5.4
Urogenital/Gynecological	2.7	4.5	1.9	0.0	0.0	0.0	4.5	14.3	2.7
Neurological	1.4	0.0	1.9	0.0	0.0	0.0	2.3	0.0	2.7
Ophtalmological/Otological	2.7	0.0	3.8	3.3	0.0	6.7	2.3	0.0	2.7
**Severity**									
Minor	68.9	68.2	69.2	66.7	73.3	60.0	70.5	57.1	73.0
Moderate	31.1	31.8	30.8	33.3	26.7	40.0	29.5	42.9	27.0
Severe	0.0	0.0	0.0	0.0	0.0	0.0	0.0	0.0	0.0

### Acute Injuries

A total of 47 acute injuries were reported by 31 athletes. 58.1% presented with one acute injury, 35.5% two, 3.2% three and 3.2% four acute injuries. The proportion of athletes with at least one acute injury did not significantly vary according to sex or discipline (Table 1).

The average weekly prevalence of acute injuries was significantly higher in explosive than endurance disciplines for all and female athletes (*p* < 0.002), while not for male athletes, and did not significantly vary between sex (Table 2). The average weekly prevalence of substantial acute injuries did not significantly vary between sex and disciplines (Table 2).

Most reported acute injuries were located at the trunk (19.1%), the hamstring (17.1%) or the knee (17.1%), affected muscles (61.7%), or ligamentous structures (14.9%), and led to one week of absence in sport (46.8%) (Table 4).

**Table 4:** Characteristics of reported acute and overuse injuries [results are presented in numbers (percentage)].

	**All athletes**										**Male athletes**														**Female athletes**													
	**Total**		**Endurance athletes**				**Explosive athletes**				**Total**		**Endurance male athletes**						**Explosive male athletes**						**Total**		**Endurance female athletes**						**Explosive female athletes**					
	**Total**		**Acute**		**Overuse**		**Acute**		**Overuse**		**Total**		**Total**		**Acute**		**Overuse**		**Total**		**Acute**		**Overuse**		**Total**		**Total**		**Acute**		**Overuse**		**Total**		**Acute**		**Overuse**	
**TOTAL**	**117**	**(100.0)**	**9**	**(100.0)**	**24**	**(100.0)**	**38**	**(100.0)**	**46**	**(100.0)**	**64**	**(100.0)**	**25**	**(100.0)**	**8**	**(100.0)**	**17**	**(100.0)**	**39**	**(100.0)**	**19**	**(100.0)**	**20**	**(100.0)**	**53**	**(100.0)**	**8**	**(100.0)**	**1**	**(100.0)**	**7**	**(100.0)**	**45**	**(100.0)**	**19**	**(100.0)**	**26**	**(100.0)**
**LOCATION**																																						
Head	1	(0.9)	0	(0.0)	0	(0.0)	1	(2.6)	0	(0.0)	0	(0.0)	0	(0.0)	0	(0.0)	0	(0.0)	0	(0.0)	0	(0.0)	0	(0.0)	1	(1.9)	0	(0.0)	0	(0.0)	0	(0.0)	1	(2.2)	1	(5.3)	0	(0.0)
Trunk	15	(12.8)	1	(11.1)	0	(0.0)	8	(21.1)	6	(13.0)	9	(14.1)	0	(0.0)	0	(0.0)	0	(0.0)	9	(23.1)	6	(31.6)	3	(15.0)	6	(11.3)	1	(12.5)	1	(100.0)	0	(0.0)	5	(11.1)	2	(10.5)	3	(11.5)
Upper extremity	9	(7.7)	1	(11.1)	0	(0.0)	3	(7.9)	5	(10.9)	7	(10.9)	1	(4.0)	1	(12.5)	0	(0.0)	6	(15.4)	3	(15.8)	3	(15.0)	2	(3.8)	0	(0.0)	0	(0.0)	0	(0.0)	2	(4.4)	0	(0.0)	2	(7.7)
Pelvic/Hip/Groin	12	(10.3)	1	(11.1)	4	(16.7)	4	(10.5)	3	(6.5)	5	(7.8)	2	(8.0)	1	(12.5)	1	(5.9)	3	(7.7)	2	(10.5)	1	(5.0)	7	(13.2)	3	(37.5)	0	(0.0)	3	(42.9)	4	(8.9)	2	(10.5)	2	(7.7)
Quadriceps	5	(4.3)	0	(0.0)	0	(0.0)	3	(7.9)	2	(4.3)	2	(3.1)	0	(0.0)	0	(0.0)	0	(0.0)	2	(5.1)	0	(0.0)	2	(10.0)	3	(5.7)	0	(0.0)	0	(0.0)	0	(0.0)	3	(6.7)	3	(15.8)	0	(0.0)
Hamstrings	26	(22.2)	1	(11.1)	2	(8.3)	7	(18.4)	16	(34.8)	14	(21.9)	3	(12.0)	1	(12.5)	2	(11.8)	11	(28.2)	3	(15.8)	8	(40.0)	12	(22.6)	0	(0.0)	0	(0.0)	0	(0.0)	12	(26.7)	4	(21.1)	8	(30.8)
Knee	14	(12.0)	2	(22.2)	6	(25.0)	6	(15.8)	0	(0.0)	10	(15.6)	7	(28.0)	2	(25.0)	5	(29.4)	3	(7.7)	3	(15.8)	0	(0.0)	4	(7.5)	1	(12.5)	0	(0.0)	1	(14.3)	3	(6.7)	3	(15.8)	0	(0.0)
Anterior lower leg	5	(4.3)	0	(0.0)	3	(12.5)	0	(0.0)	2	(4.3)	4	(6.3)	3	(12.0)	0	(0.0)	3	(17.6)	1	(2.6)	0	(0.0)	1	(5.0)	1	(1.9)	0	(0.0)	0	(0.0)	0	(0.0)	1	(2.2)	0	(0.0)	1	(3.8)
Posterior lower leg	16	(13.7)	2	(22.2)	3	(12.5)	3	(7.9)	8	(17.4)	5	(7.8)	3	(12.0)	2	(25.0)	1	(5.9)	2	(5.1)	0	(0.0)	2	(10.0)	11	(20.8)	2	(25.0)	0	(0.0)	2	(28.6)	9	(20.0)	3	(15.8)	6	(23.1)
Ankle (lateral)	2	(1.7)	0	(0.0)	0	(0.0)	2	(5.3)	0	(0.0)	1	(1.6)	0	(0.0)	0	(0.0)	0	(0.0)	1	(2.6)	1	(5.3)	0	(0.0)	1	(1.9)	0	(0.0)	0	(0.0)	0	(0.0)	1	(2.2)	1	(5.3)	0	(0.0)
Foot	12	(10.3)	1	(11.1)	6	(25.0)	1	(2.6)	4	(8.7)	7	(10.9)	6	(24.0)	1	(12.5)	5	(29.4)	1	(2.6)	1	(5.3)	0	(0.0)	5	(9.4)	1	(12.5)	0	(0.0)	1	(14.3)	4	(8.9)	0	(0.0)	4	(15.4)
**TYPE**																																						
Concussion	1	(0.9)	0	(0.0)	0	(0.0)	1	(2.6)	0	(0.0)	0	(0.0)	0	(0.0)	0	(0.0)	0	(0.0)	0	(0.0)	0	(0.0)	0	(0.0)	1	(1.9)	0	(0.0)	0	(0.0)	0	(0.0)	1	(2.2)	1	(5.3)	0	(0.0)
Stress fracture	2	(1.7)	0	(0.0)	2	(8.3)	0	(0.0)	0	(0.0)	0	(0.0)	0	(0.0)	0	(0.0)	0	(0.0)	0	(0.0)	0	(0.0)	0	(0.0)	2	(3.8)	2	(25.0)	0	(0.0)	2	(28.6)	0	(0.0)	0	(0.0)	0	(0.0)
Other bone injuries	9	(7.7)	1	(11.1)	3	(12.5)	1	(2.6)	4	(8.7)	5	(7.8)	4	(16.0)	1	(12.5)	3	(17.6)	1	(2.6)	1	(5.3)	0	(0.0)	4	(7.5)	0	(0.0)	0	(0.0)	0	(0.0)	4	(8.9)	0	(0.0)	4	(15.4)
Ligmanetous injuries	9	(7.7)	0	(0.0)	1	(4.2)	7	(18.4)	1	(2.2)	5	(7.8)	1	(4.0)	0	(0.0)	1	(5.9)	4	(10.3)	3	(15.8)	1	(5.0)	4	(7.5)	0	(0.0)	0	(0.0)	0	(0.0)	4	(8.9)	4	(21.1)	0	(0.0)
Meniscus/cartilage	3	(2.6)	0	(0.0)	0	(0.0)	2	(5.3)	1	(2.2)	1	(1.6)	0	(0.0)	0	(0.0)	0	(0.0)	1	(2.6)	1	(5.3)	0	(0.0)	2	(3.8)	0	(0.0)	0	(0.0)	0	(0.0)	2	(4.4)	1	(5.3)	1	(3.8)
Muscle strain/tear	15	(12.8)	4	(44.4)	0	(0.0)	11	(28.9)	0	(0.0)	8	(12.5)	4	(16.0)	4	(50.0)	0	(0.0)	4	(10.3)	4	(21.1)	0	(0.0)	7	(13.2)	0	(0.0)	0	(0.0)	0	(0.0)	7	(15.6)	7	(36.8)	0	(0.0)
Muscle cramps	59	(50.4)	2	(22.2)	8	(33.3)	12	(31.6)	37	(80.4)	30	(46.9)	5	(20.0)	1	(12.5)	4	(23.5)	25	(64.1)	8	(42.1)	17	(85.0)	29	(54.7)	5	(62.5)	1	(100.0)	4	(57.1)	24	(53.3)	4	(21.1)	20	(76.9)
Tendinosis/tendinopathy/ aponeurosis	15	(12.8)	1	(11.1)	10	(41.7)	2	(5.3)	2	(4.3)	12	(18.8)	10	(40.0)	1	(12.5)	9	(52.9)	2	(5.1)	1	(5.3)	1	(5.0)	3	(5.7)	1	(12.5)	0	(0.0)	1	(14.3)	2	(4.4)	1	(5.3)	1	(3.8)
Arthritis/synovitis/bursitis/ impignement	4	(3.4)	1	(11.1)	0	(0.0)	2	(5.3)	1	(2.2)	3	(4.7)	1	(4.0)	1	(12.5)	0	(0.0)	2	(5.1)	1	(5.3)	1	(5.0)	1	(1.9)	0	(0.0)	0	(0.0)	0	(0.0)	1	(2.2)	1	(5.3)	0	(0.0)
**SEVERITY**																																						
Minor	62	(53.0)	5	(55.6)	14	(58.3)	17	(44.7)	26	(56.5)	34	(53.1)	15	(60.0)	4	(50.0)	11	(64.7)	19	(48.7)	8	(42.1)	11	(55.0)	28	(52.8)	4	(50.0)	1	(100.0)	3	(42.9)	24	(53.3)	9	(47.4)	15	(57.7)
Moderate	36	(30.8)	1	(11.1)	6	(25.0)	12	(31.6)	17	(37.0)	21	(32.8)	6	(24.0)	1	(12.5)	5	(29.4)	15	(38.5)	8	(42.1)	7	(35.0)	15	(28.3)	1	(12.5)	0	(0.0)	1	(14.3)	14	(31.1)	4	(21.1)	10	(38.5)
Severe	19	(16.2)	3	(33.3)	4	(16.7)	9	(23.7)	3	(6.5)	9	(14.1)	4	(16.0)	3	(37.5)	1	(5.9)	5	(12.8)	3	(15.8)	2	(10.0)	10	(18.9)	3	(37.5)	0	(0.0)	3	(42.9)	7	(15.6)	6	(31.6)	1	(3.8)

*For all athletes, there was a significant difference between acute and overuse injuries in the distribution of injury type (Chi2 = 40.9; p < 0.0001), but not for location and type*.

The main injury diagnoses of acute injuries were lower leg strain/tear in male endurance athletes (25%), trunk muscle cramps/spasms in female endurance athletes (100%, corresponding to the only one acute injury for female endurance athletes) and male explosive athletes (31.6%), and hamstring strain/tear in female explosive athletes (21.1%).

### Overuse Injuries

A total of 70 overuse injuries were reported by 37 athletes. 48.6% presented with one overuse injury, 29.7% two, 5.4% three, and 16.2% four overuse injuries. The proportion of athletes with at least one overuse injury did not significantly vary according to sex or discipline (Table 1).

The average weekly prevalence of overuse injuries and substantial overuse injuries was significantly higher in female than male athletes for endurance disciplines (*p* < 0.0001), and higher in endurance disciplines when compared to explosive disciplines for all, male and female athletes (*p* < 0.001; Table 2).

Most reported overuse injuries were located at the hamstring (25.7%), the posterior lower leg (15.7%) or the foot (14.3%), in most cases affected muscles (64.3%), and led to 1 week of absence from sport (57.1%) (Table 4).

The main injury diagnoses of overuse injuries were knee tendinopathy in male endurance athletes (29.4%), lower leg muscle cramps in female endurance athletes (28.6%), and hamstring muscle cramps/spasms in both male explosive athletes (40.0%) and female explosive athletes (21.1%).

## Discussion

The main findings of the present study were that (1) almost ninety percent of Youth and Junior Track & Field (athletics) athletes presented with at least one health problem during the season: 60% an illness and 77% an injury; (2) for an average week, almost one third of Youth and Junior athletes presented with a health problem, and 11% a substantial health problem; (3) average prevalence varied significantly between endurance and explosive disciplines: higher prevalence of health problems and overuse injuries in endurance disciplines, and higher prevalence of acute injuries in explosive disciplines; (4) upper respiratory tract problems were the most commonly reported illnesses regardless of sex and disciplines; and (5) characteristics of acute and overuse injuries differed according to sex, discipline and injury type: trunk, hamstring and lower leg muscle injuries being the most commonly reported injuries. Such information is of great interest to better understand health problems in athletes and to help direct injury and illness prevention strategies (van Mechelen et al., 1992; Jacobsson et al., 2013; Edouard et al., 2015a).

Our present study confirms that Youth and Junior track and field athletes present a risk of sustaining a health problem, since almost all included athletes have presented with at least one health problem during the season (only nine athletes reported being fully healthy for the duration of the study). Moreover, health problems represent a part of an athlete's daily life since on average approximately one third of Youth and Junior athletes reported having at least one health problem each week. This is in agreement with previous studies in other sports reported in similar age-category population using the same methodology (Pluim et al., 2016; Moseid et al., 2018). A mean weekly prevalence of health problems of 21 and 43% have been reported in Dutch Junior Tennis players (Pluim et al., 2016) and in high level Norway athletes (Moseid et al., 2018), respectively. These values in Youth and Junior athletes are also comparable to those reported in adults in Norwegian Olympic level athletes (36%) (across a variety of sports) (Clarsen et al., 2013). These results reveal that health problems are frequent in such population of young athletes. This raises some questions about their future: Do these problems lead to stopping sport? What are the negative consequences of these rates of injury and illness? This is an issue that needs to be addressed. Efforts should be made to develop better understanding of the extent of the problems through epidemiological studies, and to limit this risk by preventive measures evaluated through interventional studies.

We reported no sex-related differences in the average prevalence across all health problems (except for endurance disciplines). This is in contrast with results from several different sports in youth elite athletes reporting higher health problems in girls, probably caused by higher illness prevalence (Moseid et al., 2018). In agreement with the latter result, we reported that the proportion of athletes with health problems was significantly higher in female than male athletes. This difference was probably due to the significant higher proportion of female athletes with at least one illness. Hence, this could suggest a predisposition of youth female athletes to illnesses, and especially urogenital/gynecological problems as reported in our study, and in agreement with Edouard et al. (2019). It is therefore necessary for medical teams to make provision to manage these conditions and to develop preventive measures.

An average weekly prevalence of illness of 6.8% was reported in our study, which is in agreement with results reported in Dutch Junior Tennis players (5.8%) (Pluim et al., 2016) and slightly lower than in high-level Norwegian athletes (12%) (Moseid et al., 2018). Reported illness problems were comparable with those reported in Tennis (Pluim et al., 2016): upper respiratory tract infections being the most common problem. This is also in agreement with previous study on elite adult athletes during the context of international athletics championships (Edouard et al., 2019). Strategies for prevention of upper respiratory tract infections is a priority target in athletics.

Our results reported discipline-related differences in average prevalence of health problems. Specifically a higher prevalence of all and substantial health problems and all and substantial overuse injuries in endurance disciplines, which differed from Moseid et al. (2018) reporting no differences. We also reported higher prevalence of acute injuries in explosive disciplines, which is consistent with results from Moseid et al. (2018) These results suggest that disciplines with different physical, mechanical, technical and psychological demands (Edouard et al., 2011, 2015a,b; Feddermann-Demont et al., 2014), lead to different constraints, and consequently to different injury characteristics, with acute injuries being more frequent in explosive disciplines and overuse in endurance disciplines.

Although using differing methodology, our results were similar to those from Jacobsson et al. (2013) in terms of injury proportion, proportion of overuse injuries, and injury location and type. Hamstring strains/cramps/spasms were the main injuries in athletes participating in explosive disciplines, as previously reported in youth athletes (Edouard et al., 2012; Jacobsson et al., 2013; Opar et al., 2014), and in high-level adult athletes during international athletics championships (Edouard et al., 2016). This is probably due to the important role of hamstring muscles in sprint acceleration performance (Morin et al., 2015), which makes these muscles at high risk of injury in explosive disciplines. This clearly makes hamstring injury an important target for injury prevention in athletics (Edouard et al., 2016). As Jacobsson et al. (2013), we reported few stress fractures (1.7% of all injuries), and only in endurance female athletes, in contrast with Bennell and Crossley (1996). This could be due to misdiagnosis, better prevention, or study design (e.g., Bennell and Crossley, 1996) focused their study on stress fractures).

Overuse injuries represented the health problem with the highest average weekly prevalence, in comparison to acute injuries and illnesses. This is consistent with previous findings in youth athletes and from other sports: 78% orienteers (Von Rosen et al., 2016), 47% in tennis (Pluim et al., 2016), 37% in elite Norwegian young athletes. This reinforces the need for using a surveillance system capable of capturing overuse injuries (Bahr, 2009; Clarsen et al., 2013, 2014; Pluim et al., 2016), to better understand these problem and how to prevent them.

The first strength of the study is providing data on injuries and illnesses in young athletes practicing athletics, given the lack of data in this field (Jacobsson et al., 2013; Edouard et al., 2015a). In addition, the current study is, to the best of our knowledge, the first study using the OSTRC Questionnaire to monitor injury and illness prevalence in high level Youth & Junior level track and field athletes. Indeed, we used an epidemiological methodology which takes into account the limits of the traditional “time loss” model (Bahr, 2009; Clarsen et al., 2013, 2014; Pluim et al., 2016). Then, in an individual sport like Athletics, in which data collection is a challenge (Edouard et al., 2014), almost all the targeted population participated in the study (92%) and a high response rate was sustained throughout.

Some limitations related to the data collection methods have been previously discussed (Clarsen et al., 2014; Pluim et al., 2016; Von Rosen et al., 2016; Aasheim et al., 2018; Moseid et al., 2018), it is however of interest to acknowledge them again. Since the data was reported directly by the athletes (athlete's self-reports), the quality of the data depends on their will and time, in addition to other parameters influencing self-reported data collection (Jacobsson et al., 2013; Clarsen et al., 2014; Pluim et al., 2016; Von Rosen et al., 2016; Aasheim et al., 2018; Moseid et al., 2018). Athletes can sometimes report as injury a “normal” pain related to the athletics practice, or as illness a “transient” problem (e.g., dizziness, tiredness, etc.; Pluim et al., 2016; Aasheim et al., 2018; Moseid et al., 2018). In order to limit this bias, the Athletics Ireland Physiotherapist checked and classified each health problem, and this is why we also used the “substantial problem” definition, which filters out the least consequential problems and may provide a better estimate of the impact of injuries and illnesses on athletes' health (Moseid et al., 2018). Some could have omitted or minimized their health problems, because they feared that this would have consequences for their selection in the national team. Athletes' self-reports are also dependent on both high responses rates throughout the course of the study. In the present study, the average response rate decreased as the season progressed (≈25% decrease from week 2 to week 30), without rupture at a specific period. This has also previously been reported, and it seems of great interest to better understand why the response rate decreased, for instance: Did the athletes find it boring to answer the questionnaire and did not see any interest in them? Were there explanations related to the sport: they did not want their medical information to be known at the proximity of important competitions for fear of not being selected…? Then, measures should be found to limit this missing data source leading to potential bias. No athlete was excluded because of “recurrent” missing data, which can represent a limit. Most of the data was self-reported data, limited objective data was collected, and no anthropometric data (Jacobsson et al., 2013). The questionnaire used in the present study (Oslo Sports Trauma Research Center questionnaire) has been developed and validate for use in adult athletes (Clarsen et al., 2013, 2014), and not for use in Youth athletes, which could represent a limitation (Pluim et al., 2016). However, it has previously been used with success in population of youth athletes in elite junior tennis (Pluim et al., 2016), in adolescent elite orienteerers (Von Rosen et al., 2016), in youth football players (Leppänen et al., 2019), in junior handball players (Aasheim et al., 2018), and in youth elite athletes in multiple sports (Moseid et al., 2018), and appears of interest in sports who have limited access to medical personnel (e.g., athletics; Edouard et al., 2014; Leppänen et al., 2019), and the present study population aged from 16 to 18 which are close to adults. A recall bias could lead to bias (Moseid et al., 2018), although asking on health problem occurred during the previous week aimed to limit this bias. No exposure data was collected (training and competition duration and intensity), which could have been of interest to better understand injury risk. We did not analyse subsequent or recurrent health problems. No power calculations were performed to establish the size of the study population as a basis for statistical testing, since we aimed to reach the entire population defined elite track and field athletes in Ireland.

The relatively high prevalence of health problems in this group of Youth and Junior track and field athletes is a cause for concern.

Prevention is key and can be done through education of athletes and all their stakeholders (coaches, parents, medical teams, athlete's caregivers; Jacobsson et al., 2018). Education programmes should take into account the knowledge of targeted audiences and characteristics of health problems being addressed (Jacobsson et al., 2018; Rodríguez-serrano et al., 2018). Stakeholders should pay attention and not neglect athletes' complaints of pain or fatigue, and where appropriate early care of health problems should be facilitated (including a clear diagnosis and an optimized treatment/care or rehabilitation). All key stakeholders should be involved in the development of primary prevention interventions (Jacobsson et al., 2013; Pluim et al., 2016).

For illness, prevention measures could include: screening tests for airway problems, but also general illness prevention measures (including but not restricted to; Engebretsen et al., 2010; Hanstad et al., 2011; Alonso et al., 2012; Périard et al., 2017; Timpka et al., 2017; Moseid et al., 2018): drinking regularly and only “safe” water, eating only “safe” food, regular hand washing, decreasing contact with sick people, avoiding dehydration & heat stress, and increasing uptake of vaccinations. Time should be spent on developing adolescent-adapted education resources, focused on eating, sleeping, social media, and screen time.

For injuries, prevention measures should be focused on muscle injuries, especially located on the hamstring, calf and trunk, by general strengthening/stretching programmes, screening athletes at risk, and providing individualized deficiency-based programmes. Efforts should also be made to better understand the risk factors and mechanisms of these main injuries to develop/improve prevention measures.

## Conclusions

This study provides important information regarding the extent of health problem in Youth and Junior track and field athletes. Almost all Youth and Junior athletes presented at least one health problem during the season, and almost a third a health problem at each time of the season. Hamstring, trunk, and lower leg muscle injuries were the most frequent reported injuries, and upper respiratory tract problems the most frequently reported illnesses. These results could help orient injury and illness prevention strategies in Youth and Junior athletes toward these main injuries.

## Data Availability

The datasets analyzed in this manuscript are not publicly available. Requests to access the datasets should be directed to paulcarragher@instituteofsport.ie.

## Ethics Statement

The studies involving human participants were reviewed and approved by the study was approved by the Saint-Etienne University Hospital Ethical Committee (IORG0004981). Written informed consent to participate in this study was provided by the participants' legal guardian/next of kin.

## Author Contributions

PC conceived, designed the study, and performed data collection. PC, AR, and PE analyzed the data, interpreted the results and edited, critically revised the manuscript, and approved the final version. PC and PE drafted the manuscript and prepared the table/figure.

### Conflict of Interest Statement

The authors declare that the research was conducted in the absence of any commercial or financial relationships that could be construed as a potential conflict of interest.
